# Training nurses in task-shifting strategies for the management and control of hypertension in Ghana: a mixed-methods study

**DOI:** 10.1186/s12913-017-2026-5

**Published:** 2017-02-02

**Authors:** Joyce Gyamfi, Jacob Plange-Rhule, Juliet Iwelunmor, Debbie Lee, Sarah R. Blackstone, Alicia Mitchell, Michael Ntim, Kingsley Apusiga, Bamidele Tayo, Kwasi Yeboah-Awudzi, Richard Cooper, Gbenga Ogedegbe

**Affiliations:** 1 New York University School of Medicine, NYU Langone Medical Center, New York, NY 10016 USA; 2 NYU College of Global Public Health, New York University, New York, NY 10003 USA; 3 School of Medical Sciences, Kwame Nkrumah University of Science and Technology, Kumasi, Ghana; 4 University of Illinois at Urbana-Champaign, Urbana, IL 61801 USA; 5 Stritch School of Medicine, Loyola Chicago Medical Center, Maywood, IL 60153 USA; 6 Stony Brook University School of Medicine, Stony Brook, NY 11797 USA; 7 Ghana Health Service, Kumasi, Ghana

**Keywords:** Task-shifting strategies, Hypertension, Nurses, Sub-Saharan Africa, Ghana

## Abstract

**Background:**

Nurses in Ghana play a vital role in the delivery of primary health care at both the household and community level. However, there is lack of information on task shifting the management and control of hypertension to community health nurses in low- and middle-income countries including Ghana. The purpose of this study was to assess nurses’ knowledge and practice of hypertension management and control pre- and post-training utilizing task-shifting strategies for hypertension control in Ghana (TASSH).

**Methods:**

A pre- and post- test survey was administered to 64 community health nurses (CHNs) and enrolled nurses (ENs) employed in community health centers and district hospitals before and after the TASSH training, followed by semi-structured qualitative interviews that assessed nurses’ satisfaction with the training, resultant changes in practice and barriers and facilitators to optimal hypertension management.

**Results:**

A total of 64 CHNs and ENs participated in the TASSH training. The findings of the pre- and post-training assessments showed a marked improvement in nurses’ knowledge and practice related to hypertension detection and treatment. At pre-assessment 26.9% of the nurses scored 80% or more on the hypertension knowledge test, whereas this improved significantly to 95.7% post-training. Improvement of interpersonal skills and patient education were also mentioned by the nurses as positive outcomes of participation in the intervention.

**Conclusions:**

Findings suggest that if all nurses receive even brief training in the management and control of hypertension, major public health benefits are likely to be achieved in low-income countries like Ghana. However, more research is needed to ascertain implementation fidelity and sustainability of interventions such as TASSH that highlight the potential role of nurses in mitigating barriers to optimal hypertension control in Ghana.

**Trial registration:**

Trial registration for parent TASSH study: NCT01802372. Registered February 27, 2013.

## Background

Shortages in the healthcare workforce in sub-Saharan Africa (SSA) and poorly trained health workers have reached a crisis point, [1–3] limiting effective reduction of hypertension-related morbidity and mortality rates in patients. In Africa, there are 2.7 doctors and 12.4 nursing/midwifery personnel per 10,000 people, compared to 21.5 doctors and 44.9 nursing/midwifery personnel per 10,000 in North America [4]. One of the greatest challenges to meeting the need for adequate care and management of high blood pressure in Ghana is the acute shortage of healthcare workers [5]. In Ghana, there is one physician and nine nursing/midwifery personnel per 10,000 patients [4].

The shortage of health workers is partly due to migration of health personnel in search of a better standard of living, higher salaries, access to advanced technology and more stable political conditions [6]. These shortages are a multifaceted problem, and improvements are needed if reduction in hypertension related morbidity in SSA is to be achieved. In Ghana, more than 3.5 out of 15.8 million individuals over age 15 are classified as hypertensive; making hypertension the second leading cause of outpatient morbidity in adults age 45 and over [7]. Changes in diet and lifestyle which has resulted in increasing numbers of people with obesity, sedentary life styles, increased salt intake and decrease in intake of fruits and vegetables partially drives the hypertension epidemic [8].

Moreover, the rate of awareness, treatment and control of hypertension are abysmally low in this population. In a population-based survey of 714 young hypertensive adults from three urban poor communities in Accra, Ghana, Awuah and colleagues [9] observed that only 7.4% were aware of their condition, 4% were on antihypertensive medication, while only 3.5% of the hypertensive individuals had adequate blood pressure control (BP <140/90 mmHg). Such high prevalence and alarming low rates of awareness, treatment, and control underscore significant unmet need for the prevention, treatment and care of hypertension in Ghana [10]. In addition, available studies also suggest that health professionals are poorly trained in diagnosis and management of non-communicable diseases (NCDs) like hypertension and lack appropriate knowledge and skills [2, 10–12]. Thus, there is an urgent need for NCD preventative strategies in Ghana particularly community-based strategies that help in the rational use of available resources [1, 3, 12]. One such approach is a task-shifting strategy, defined as the rational distribution of primary care duties from physicians to non-physician healthcare providers such as community health nurses [5].

Task shifting to nurses is increasingly advocated as a potential solution to overcoming current shortfalls in physician populations in low- and middle-income countries like Ghana. The idea of task shifting is not entirely new. Task shifting was to be the hallmark of the WHO-led primary health care movement of the 1980s [5]. It was behind the declaration of what became known as health for all by the year 2000. For this purpose, and in order to maximize the efficient use of health workforce resources, primary care tasks are shifted from higher-trained health workers such as physicians to less highly trained health workers [5]. Despite the potential for task-shifting interventions to mitigate the NCD epidemic in low-and middle-income countries (LMICs), their effectiveness has not been widely evaluated. However, such information is needed for policy and decision makers to effectively make evidence-based recommendations for implementation of task shifting within healthcare systems in LMICs.

A cluster-randomized trial of task shifting and blood pressure in community health centers and district hospitals was undertaken in Ghana [12]. This study evaluated, among 757 hypertensive patients who receive care in community health centers (CHCs) in the Ashanti Region of Ghana, the comparative effectiveness of the implementation of the WHO Package of Essential Noncommunicable (PEN) disease interventions targeted at cardiovascular disease (CVD) risk assessment and hypertension control (intervention group), versus provision of health insurance coverage (control group), on BP reduction. Trained nurses delivered the intervention as part of Ghana’s community-based health planning and services (CHPS) program [12]. Nurses in Ghana play a vital role in delivery of primary health care at the community level. While the reliability of having nurses deliver the WHO strategy for CVD risk assessment and optimal hypertension control when compared to ‘expert’ physicians in primary care settings has been established in several LMICs [12–14], its implementation is almost non-existent in SSA [13].

In order to assess the effectiveness of task-shifting strategies, evidence of its implementation for addressing the CVD epidemic as part of existing healthcare systems in LMICs is paramount [12]. The purpose of our study is to assess the effects of the task-shifting strategy for hypertension (TASSH) on nurses’ knowledge and practice of hypertension management and control in Ghana. Specifically, we present a mixed-methods study with two primary aims: 1) assess TASSH nurses’ knowledge of hypertension and hypertension management pre- and post-TASSH training, and 2) explore nurses’ perceptions of the barriers to, and facilitators of optimal management of hypertension in Ghana. The results of this study may provide valuable information on whether the TASSH protocol improved knowledge of hypertension and management among community health nurses in the Ashanti Region of Ghana, and could potentially inform the expansion of this training program to other regions of the country.

## Methods

### Study design and participants

The cluster-randomized trial of task shifting and blood pressure control is described in detail elsewhere; [12] however, a basic overview is provided here for clarity related to the process of evaluating nurses’ knowledge and practice of hypertension management following the implementation of TASSH. Using a cluster-randomized design, 32 community health centers and district hospitals in the Ashanti region of Ghana were randomly assigned to either the intervention group (16 sites) or the control group (16 sites). A total of 757 patients with uncomplicated hypertension (systolic BP 140–179 mm Hg or diastolic BP 90–99 mm Hg and absence of target organ damage) were enrolled in this study. Study outcomes were assessed every 3 months for 1 year, and sustainability of intervention effects were evaluated at 24 months (one year after the trial is completed). Patients at the sites randomized to the control group received health insurance coverage in addition to usual care, while patients at the sites randomized to the intervention group received health insurance coverage plus the WHO PEN package delivered by trained nurses.

Two levels of nurses were trained; first level or community health nurses (CHNs) and second-level or enrolled nurses (ENs). The nurses are employed at one of the 32 sites included in the TASSH parent study [12]. In Ghana, nurses’ initial training is generally focused on basic sciences and nursing practice. There is no specific course on case management of patients with hypertension and other chronic diseases. For enrolled nurses, their knowledge of case management is received on the job and even that is very limited. As for the community health nurses, minimum hypertension management training is received on the job since they are mainly responsible for managing reproductive and maternal child health issues in rural communities. As part of the TASSH study protocol, once patients’ eligibility was confirmed, the trained nurses at the intervention sites collected data on the patients’ medical history. Next, they measured the patients’ weight, height, waist circumference, and BP with an automated device. They also performed laboratory tests using point-of-care testing to assess fasting blood glucose, cholesterol levels, and urine dipstick for proteinuria. The information obtained from the medical history, physical examination, and laboratory tests was then used by the nurses to assess patients’ CV risk with the aid of the well validated WHO prediction risk charts that are contextualized for each region [15]. Once the patient’s CV risk was determined (low, medium, or high), those with high CV risk were referred to the district hospital for further management. The trained nurse initiated the treatment protocol for patients who were at low or medium risk according to their BP level using any one of the four major antihypertensive medications—bendrofluazide, a diuretic; amlodipine, a calcium channel blocker; lisinopril, an angiotensin converting enzyme inhibitor; and metoprolol, a beta-blocker [12].

### Task-shifting strategy for hypertension control (TASSH): nurse’s training

Training procedures were centralized in Ghana and began in August 2012 and then every 6 months during patient recruitment and follow-up, for an average of four training sessions. All study staff and nurses received training on the study protocol, including the WHO CVD risk assessment package [16], techniques for collecting anthropometric data, BP measurements, questionnaire administration, blood draw, urine collection, and processing techniques, which were standardized between health facilities. Study nurses received additional training in behavioral counseling techniques using motivational interviewing (MI), which is a strategy to help patients work through ambivalence with their health condition and commit to change; MI techniques can be applied to management of several diseases, including hypertension [17, 18]. Prior to the TASSH training, many of the nurses had not received training in the WHO CVD risk assessment and MI techniques, thus augmenting previous training received during their nursing certification programs. The nurses’ training was conducted in person by study principal investigators whom are both clinical hypertension specialist and two experienced masters level study staff trained in epidemiology and the biomedical sciences with clinical backgrounds. There were onsite supervision by the study coordinators and periodic onsite booster training was conducted. Project coordinators and nurses received training in proper procedures for obtaining informed consent from all study participants and study data collection. The study nurses were responsible for screening, and referring patients with complicated hypertension to a specialist; assisting with data collection as necessary, and conducting the MI counseling sessions with patients. All field staff involved in data collection were trained before patient data collection began. All participants of the study provided verbal and written informed consent prior to initiating any study activities. The study was approved by the Institutional Review Board of New York University School of Medicine and the Committee for Human Research, Publications and Ethics at Kwame Nkrumah University of Science and Technology, Kumasi, Ghana.

### Quantitative data collection and analyses

For this particular study on nurses training, a mixed-method, pre–posttest survey design was used. The research team provided a modified hypertension evaluation of lifestyle and management (HELM) questionnaire [19], 19 multiple choice questions) to nurses involved in the TASSH hypertension training program (see Appendix). The HELM questionnaire was modified to include mostly ‘Yes/No’ responses; and we also included questions on MI and nurses’ confidence level in the management of hypertension. Fifty-two nurses agreed to participate in the pre –assessment (which was completed prior to the training); and the post assessment was completed after 4 consecutive TASSH trainings or 2 years from initial training. Forty- nine nurses completed the post-assessment. The questionnaire evaluated nurses’ education, and current hypertension knowledge with questions such as; interpretation of systolic and diastolic blood pressure numbers, ultimate consequences/comorbidities of persistent hypertension, lifestyle changes for hypertension prevention, motivational interviewing technique, and their confidence in detecting and treating hypertension. The questionnaire was administered to assess knowledge of material presented in the TASSH training. The research team did not assess practices with the quantitative questionnaire, but focused solely on knowledge retention from initial training. Statistical data analyses were performed using SPSS software (version 22.0). Descriptive statistics were presented as mean scores, standard deviations and percents; and they describe the demographic characteristics of the participants along with other study variables. The questionnaire was scored by assigning 1 point for each correct answer out of 19 questions. Multiple linear regression was used to determine if there were associations between nurses’ characteristics (e.g. rural/urban, training received) and test scores. Paired sample *t*-test was used to compare the pre- and post- training results.

### Qualitative data collection and analysis

Semi-structured interviews were conducted with 38 (73%) of the participating nurses (by a trained facilitator) and we also assessed the nurses’ personal experiences with TASSH, satisfaction with training, and barriers and facilitators to implementing hypertension-related interventions in the healthcare settings. Specifically, we asked the following questions: overall, what knowledge have you gained from the TASSH training program? What are the facilitators to hypertension care in your facility? What are the barriers to hypertension care in your facility? Based on your interaction with your patients, what do they describe as challenges to accessing appropriate care?. Thematic analysis following guidelines as described by Braun & Clarke [20] was used to identify recurring patterns and themes within the qualitative interview data. This was conducted by two members of the research team. Any discrepancies were resolved by consulting a third member of the team who reviewed the transcripts and coding process, and worked with the team members to reach a consensus. Following familiarity with the data, similar statements were identified and allocated codes. We then placed the codes into an Excel spreadsheet so as to search for themes. Early themes that emerged where what nurses gained from participating in TASSH. Due to the exploratory nature of this study, we used an inductive qualitative approach, without the presupposition of an existing theoretical framework [21]. The themes were further reviewed and analyzed by researchers to ensure that the statements were suitably placed to support the themes and that all significant data were fully captured in the analysis.

## Results

### Quantitative findings

Characteristics of the nurses are summarized in Table 1. A total of 64 nurses participated in this study, of which 28 are community health nurses (CHNs) and 35 are enrolled nurses (ENs). One nurse is a staff nurse, which is equivalent to a registered nurse in the United States. Majority (87.5%) of the participants were female.

**Table 1:** Characteristics of nurses involved in TASSH training

Characteristics	Number	Mean (SD)	Percent
Age	44	30.55 (6.68)	--
Female	56	--	87.5
CHN	28	--	43.8
EN	35	--	54.7
Years out of school	46	5.96 (6.82)	--
Years of work experience	60	4.44 (5.33)	--
Employed at urban site	32	--	50
Employed at rural site	32	--	50
Health care facilities’ annual patient load	32	49404 (50262.49)	--
Pre-test score	52	11.65 (3.96)	--
Post-test score	47	17.09 (1.28)	--
Score change	39	5.97 (4.34)	--
^a^Proficiency at pre-test	52	--	26.9
^a^Proficiency at post-test	47	--	95.7

^a^Percent of nurses with 80% score or more on the hypertension Assessment

Fifty percent of the nurses were employed at rural facilities. The mean age for the participants is 30.6 years old ± 6.68, with 4.4 years of nursing experience. On average, nurses completed their formal nursing education about 5.9 years ago. Less than half of the nurses received prior training in anthropometric measurements (45.3%) and basic laboratory procedures (48.4%). Of the 64 nurses, 76.6% (*n* = 47) completed the hypertension knowledge assessment at the post-test time point. The mean score on the pre- and post-test was 11.65 ± 3.95 and 17.09 ± 1.2 respectively. At baseline, 26.9% (*n* = 14) of the nurses were considered to be proficient at hypertension management, and at post-test 95.7% (*n* = 45) were considered proficient. Proficiency was determined by an 80% score on the hypertension knowledge assessment and was coded as 0 = not proficient and 1 = proficient. The assessment indicated improvements in the nurses’ hypertension knowledge post study training; however these improvements were not statistically significant. The intervention and control groups each had 32 nurses from the 16 sites (two nurses per site). There was no differences in the mean assessment scores between the intervention and control groups (*p* = 0.252). The level of occupation (i.e. CHN or EN) did not have a significant effect on pre- or post-test score, or on overall score improvement (Table 2).

**Table 2:** Regression effects of nurses characteristics on hypertension knowledge assessment scores

	Pre-test Score	Post-test Score
Covariates	*β (Standard Error)*	*β (Standard Error)*
Group	−1.057 (1.404)	-.828 (.416)
Gender	.945 (1.866)	.122 (.587)
Advanced Lab Training	5.364 (2.238)*	.433 (.659)
Pharmacy Training	3.182 (2.516)	.283 (.539)
Nurses Confidence	−1.462 (1.316)	.087 (.380)
Urban	1.118 (1.439)	-.738 (.418)
Occupation	.878 (1.473)	-.397 (.426)

*p < .001

Prior professional training in laboratory procedures did not have a significant effect on score improvement. For instance, enrolled nurses were more likely to be trained in blood draws (*χ*2 = 64; *p* < .001), however, this did not improve post-test scores for this group. Overall, pre-and post-test results and proficiency were not affected by the following factors: nurses’ group assignment (intervention or control), prior on the job training in hypertension diagnosis and appropriate drug treatment, laboratory procedures, and location of job (rural/urban) or nurses’ confidence level in managing hypertension.

### Qualitative findings

Despite a lack of statistical significant quantitative effects of the intervention on test scores, nurses noted positive outcomes with respect to the TASSH training in the qualitative interviews. There were numerous mentions by the nurses that their ability to manage hypertension was better after the intervention, and that their knowledge of interpreting measurements and patient labs improved, particularly interpretation of the WHO prediction chart for CVD risk estimation. Improvement of interpersonal skills and patient education were also mentioned as positive outcomes of participation in the intervention. Knowledge of factors that cause hypertension and methods of controlling hypertension were noted as well. Nurses’ perceptions of the training are reflected in the following comments;

“Since doing the training, I am able to counsel patients on control and treatment of hypertension by discussing diet and lifestyle changes and their goals. I also educate patients about their medications.”

“It has been helpful to identify and access patients with hypertension, read their lab results with them, accurately take their blood pressure measurements, while using the risk prediction chart for estimation of CVD risk.”

The nurses also discussed potential barriers related to managing and controlling hypertension effectively, which occur at the patient, provider and systems level. At the patient level, lack of money for transportation, lack of and costs of medication, and non-medication adherence were reported to adversely impact hypertension care. One nurse stated the following:

“Our patients lack money and insurance, then the cost of medications and lack of transportation to our facility makes it difficult to help them manage their hypertension.”

At the provider and systems level, nurses noted that their workload alongside, suboptimal staffing, inadequate equipment and space for proper patient care, and language barriers between providers and patients limit their capacity to adequately contribute significantly to the management of blood pressure. Others discussed poor follow up *visits* with patients, *and a lack of patient motivation* as potential barriers. In terms of facilitators, some nurses were of the opinion that; “a hypertension clinic,” with “adequate equipment,” “staffing,” and opportunity for “scheduling follow up care” while “providing education on hypertension to all patients,” will help decrease the number of cases in the region.

## Discussion

This mixed-methods study is part of a larger study in Ghana evaluating the effectiveness of interventions targeted at blood pressure control in the Ashanti region. The delivery of strategies such as counseling on lifestyle modification or initiation of treatment by well trained and knowledgeable non-physician health care workers such as CHNs is well documented and has shown to be highly cost-effective in the management of other conditions such as diabetes, and HIV/AIDS [22–25]. Prior to the TASSH training, some nurses had limited knowledge of performing basic tasks such as anthropometric measurements or categorizing patients into high, medium, or low-risks for CVD based on given cholesterol levels, age, gender, smoking status, systolic BP and diabetes status. Similar knowledge gaps have been demonstrated in assessment of non-physician providers and other healthcare providers caring for HIV patients in a Cameroon rural hospital and a Nigerian teaching hospital, respectively [26, 27]. In both of these studies, providers in the higher professional grades (based on years of education and level of training) performed better on knowledge questionnaires.

The TASSH training resulted in improvements in the hypertension knowledge among nurses in both the intervention and control group, although the effects were not statistically significant in relation to whether the nurses were CHNs or ENs. More ENs reported receiving prior training in blood draw as they are stationed at the health facility and that skill is required to effectively carry out their responsibilities, whereas CHNs are charged with providing preventative care in remote communities. The implications of these findings are broad particularly as nurses are the largest group of healthcare workers in Ghana. Our findings suggest that training nurses in hypertension management can increase their knowledge of treatment and control strategies which may decrease hypertension rates in the country. Though these findings were not statistically significant, we did see an increase in proficiency of hypertension management from pre- to post-test (26.9 to 95.7%, respectively). Furthermore, we saw a mean increase of more than five points between pre- and post-test scores, indicating improved knowledge of hypertension between the two time points. Qualitative findings support this assertion as nurses reported positive outcomes with the training including increased knowledge and skills in MI techniques which may enable them to provide counseling on prevention and control of hypertension to patients post training. Many of the nurses also made reference to improvements in their ability to predict CVD risk using the WHO CVD-risk prediction charts and accurately measure blood pressure post- training; with enhanced skills to initiate treatment, monitor and document adverse drug reaction and sustain a discussion on the consequences of poor management of hypertension with patients. Nurses became proficient in identifying complicated hypertension cases and made appropriate patient referrals to hypertension specialists post TASSH training. The confidence of the nurses to effectively identify and treat uncomplicated hypertensive patients also improved post TASSH training. These findings are similar to other task shifting studies conducted in SSA. For example, Coleman and colleagues [28] showed that introduction of specific treatment protocols for NCDs to selectively-trained nurses in a largely rural district in South Africa enabled control of 68% of patients with hypertension, 82% with diabetes, and 84% with asthma.

Although the TASSH program is promising, there are barriers and challenges as discussed by the nurses worth noting that are likely to have larger impact on the continued prevalence of hypertension in the region, if not addressed. These challenges occur at the healthcare system, provider, and patient level and may prevent the efficient delivery of strategies aimed at enabling optimal management and control of hypertension [12]. Nurses indicated that barriers to hypertension management at the patient level are partly determined by the extent of financial resources which negatively influences whether patients purchase their medications, or adhere to treatment, or even visit the health center. These negative effects of patient barriers have been found in other studies. One study among 161 hypertensive patients in Dar es Salaam, Tanzania, cited cost of treatment among the reasons mentioned most often for not seeking health care [29]. In Ghana, Buabeng and colleagues [30] found that in a sample of 128 patients, 93% did not comply with their medications with majority citing unaffordable drug prices as the main reason for non-compliance. At the systems level, nurses noted the following barriers: lack of access to adequate space and suitable waiting and exam rooms for proper patient care, which hinders patient confidentiality; lack of technology and adequate diagnostic equipment; non-functional comprehensive laboratory; lack of infection control protocols; and inefficient patient record keeping system which limit the nurses capacity to adequately care for the patients. Clearly, the cost associated with rectifying these issues are beyond the current capacity of Ghana’s national health system; however, these matters should be on the Ministry of Health’s agenda. Similar findings have also been observed in Nigeria [31]. At the provider level, nurses mentioned challenges affecting professional growth and patient care, including lack of continued professional education and training, language barriers, and lack of standards and uniformity in treatment protocol. In anticipation of the above limitations, the research team provided all of the equipment needed for the training, including automatic blood pressure machines, laboratory equipment for measurements of serum and urine samples for lipids, glucose and other hypertension indicators. We also equipped nurses with medical gloves, alcohol swabs and other infection prevention equipment, as infection control measures at the various facilities proved troublesome.

There are several limitations worth noting in this study. First, this study is based on self-report with a small sample size which limits the generalizability of our findings. Further, we had limited opportunities to observe nurses as they managed or counseled non-TASSH patients with hypertension so we could not confirm what they reported. Moreover, this study only assessed changes in knowledge, and not changes in practices. As a result, the pre-training reports and the post-training improvements among our nurses must be interpreted cautiously as it is possible that the nurses responded in ways that reflected best practice rather than what they routinely do. Future studies could build upon these results by observing actual clinical practices of nurses to determine whether changes in knowledge actually equate to changes in delivery of care. Finally, our small sample size limits the statistical power of this study, which could contribute to the lack of statistically significant findings. Despite this, we found a considerable increase in the percent of nurses proficient in hypertension management from pre- to post-test and improvements in scores on the hypertension knowledge assessment.

## Conclusion

In conclusion, the findings from our study support the idea that non-physician health care workers such as CHNs occupy an important role in striving for optimal hypertension control in SSA including Ghana. Indeed, if all nurses receive even brief training in the management and control of hypertension, major public health benefits are likely to be achieved in many low-income countries struggling to cope with a double burden of infectious and chronic diseases. Evidence from this study also shows that positive interactions between nurses and patients also provides opportunities for open discussions including the potential to counsel patients on behavior and lifestyle changes in relation to hypertension prevention. There is a clear need for task-shifting strategies in LMICs [32]. Future research should consider the evaluation of interventions targeted at hypertension control, particularly in relation to observations of implementation fidelity-i.e. whether the interventions are implemented as intended [33, 34] and implementation sustainability [35, 36]-i.e. how to ensure the continued use of task shifting interventions among nurses in the community health centers beyond the initial implementation.

## Figures and Tables

**Fig. 1:**
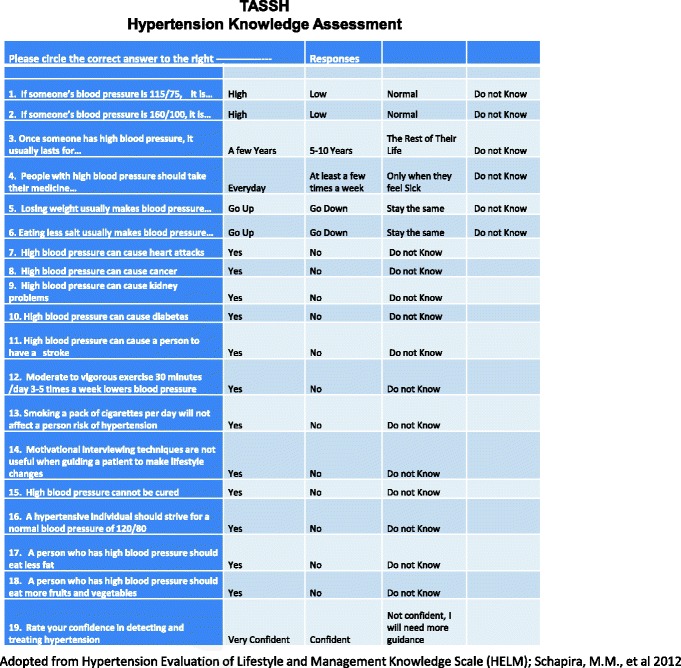
Hypertension Knowledge Assessment questionnaire

## Abbreviations

BP: Blood pressure
CHNs: Community health nurses
CHPS: Community-based health planning and services program
CVD: Cardiovascular disease
ENs: Enrolled nurses
HELM: Hypertension evaluation of lifestyle and management
LMICs: Low-and-middle income countries
NCDs: Non-communicable diseases
PEN: Package of Essential Noncommunicable
SSA: Sub-Saharan Africa
TASSH: Task-shifting strategies for hypertension control in Ghana
WHO: World Health Organization
