# Erratum to: Training nurses in task-shifting strategies for the management and control of hypertension in Ghana: a mixed-methods study

**DOI:** 10.1186/s12913-017-2161-z

**Published:** 2017-03-17

**Authors:** Joyce Gyamfi, Jacob Plange-Rhule, Juliet Iwelunmor, Debbie Lee, Sarah R. Blackstone, Alicia Mitchell, Michael Ntim, Kingsley Apusiga, Bamidele Tayo, Kwasi Yeboah-Awudzi, Richard Cooper, Gbenga Ogedegbe

**Affiliations:** 1 New York University School of Medicine, NYU Langone Medical Center, New York, NY 10016 USA; 2 NYU College of Global Public Health, New York University, New York, NY 10003 USA; 3 School of Medical Sciences, Kwame Nkrumah University of Science and Technology, Kumasi, Ghana; 4 University of Illinois at Urbana-Champaign, Urbana, IL 61801 USA; 5 Stritch School of Medicine, Loyola Chicago Medical Center, Maywood, IL 60153 USA; 6 Stony Brook University School of Medicine, Stony Brook, NY 11797 USA; 7 Ghana Health Service, Kumasi, Ghana

## Erratum

Upon publication of this article [1], it was brought to our attention that the following need to be corrected:

1. In the ‘Background’ section, the sentence: “…limiting effective reduction of hypertension-related morbidity and mortality rates in patients” should be corrected to read: “…limiting effective reduction of hypertension-related morbidity and mortality rates among patients”.
2. In the ‘Background’ section, the sentence: “A cluster-randomized trial of task shifting and blood pressure in community health centers…” should be corrected to read: “A cluster-randomized trial of task shifting and blood pressure control in community health centers…”
3. In the ‘Methods’ section, number forty-nine should be changed to forty-seven.
